# Role of BgaA as a Pneumococcal Virulence Factor Elucidated by Molecular Evolutionary Analysis

**DOI:** 10.3389/fmicb.2020.582437

**Published:** 2020-09-24

**Authors:** Masaya Yamaguchi, Moe Takemura, Kotaro Higashi, Kana Goto, Yujiro Hirose, Tomoko Sumitomo, Masanobu Nakata, Narikazu Uzawa, Shigetada Kawabata

**Affiliations:** ^1^Department of Oral and Molecular Microbiology, Osaka University Graduate School of Dentistry, Suita, Japan; ^2^Department of Oral and Maxillofacial Surgery II, Osaka University Graduate School of Dentistry, Suita, Japan

**Keywords:** *Streptococcus pneumoniae*, molecular evolutionary analysis, pneumococcal cell wall-anchoring proteins, *bgaA*, *nanA*

## Abstract

*Streptococcus pneumoniae* is a major cause of pneumonia, sepsis, and meningitis. Previously, we identified a novel virulence factor by investigating evolutionary selective pressure exerted on pneumococcal choline-binding cell surface proteins. Herein, we focus on another pneumococcal cell surface protein. Cell wall-anchoring proteins containing the LPXTG motif are conserved in Gram-positive bacteria. Our evolutionary analysis showed that among the examined genes, *nanA* and *bgaA* had high proportions of codon that were under significant negative selection. Both *nanA* and *bgaA* encode a multi-functional glycosidase that aids nutrient acquisition in a glucose-poor environment, pneumococcal adherence to host cells, and evasion from host immunity. However, several studies have shown that the role of BgaA is limited in a mouse pneumonia model, and it remains unclear if BgaA affects pneumococcal pathogenesis in a mouse sepsis model. To evaluate the distribution and pathogenicity of *bgaA*, we performed phylogenetic analysis and intravenous infection assay. In both Bayesian and maximum likelihood phylogenetic trees, the genetic distances between pneumococcal *bgaA* was small, and the cluster of pneumococcal *bgaA* did not contain other bacterial orthologs except for a *Streptococcus gwangjuense* gene. Evolutionary analysis and BgaA structure indicated BgaA active site was not allowed to change. The mouse infection assay showed that the deletion of *bgaA* significantly reduced host mortality. These results indicated that both *nanA* and *bgaA* encode evolutionally conserved pneumococcal virulence factors and that molecular evolutionary analysis could be a useful alternative strategy for identification of virulence factors.

## Introduction

*Streptococcus pneumoniae* is one of most frequently isolated bacteria from community acquired pneumonia, sepsis, and bacterial meningitis (Ishiguro et al., 2013; CDC, 2019). The phylogenetic relationship of both 16S rRNA and a core set of 136 genes indicated that this pathogen belongs to mitis group of *Streptococcus* (Kawamura et al., 1995; Richards et al., 2014). *S. pneumoniae* is capable of importing various genes including antimicrobial resistance genes via horizontal transfer from related species (Salvadori et al., 2019). In the United States of America, more than 30% of clinically isolated pneumococcal bacteria are resistant to one or more antibiotics (CDC, 2019). Currently, 23-valent pneumococcal polysaccharide and 13-valent pneumococcal-conjugated vaccines are in use in various countries. These vaccines prevent pneumococcal infections caused by vaccine-targeted serotype strains and inhibit the spread of drug resistant strains (CDC, 2019). On the other hand, the selective pressure imposed by vaccination has increased the emergence of non-vaccine serotype strains (Golubchik et al., 2012).

Recently, we applied a combination of evolutionary analysis and laboratory-based approaches to evaluate the functional significance of putative virulence factors (Yamaguchi et al., 2016, 2019a; Yamaguchi, 2018). As mutations in non-essential but important genes promote the selection of bacterial lineages in single species, genes that undergo considerable negative selection would be important for the survival and/or success of the species in its host and/or the environment. Thus, a molecular evolutionary approach enables us to estimate the contributions of bacterial proteins to species success throughout its life cycle. We have previously focused on the evolutionary selective pressures on choline-binding proteins (CBPs) in *S. pneumoniae* (Yamaguchi et al., 2019a). CBPs are localized to the cell surface by binding to phosphoryl choline on the cell wall. As cell surface proteins are easily and directly accessible to the external environment, these proteins could represent attractive antigen candidates for vaccine development. Our analysis revealed that CbpJ contributes to evasion of host neutrophil-mediated killing in pneumococcal pneumonia. This is surprising as CbpJ has no known functional domains apart from signal sequences and choline-binding repeats. While there are also other types of pneumococcal cell surface proteins, their degree of evolutionary conservation remains unknown.

For this study, we focused on different motif involved in pneumococcal cell surface localization, LPXTG, which is associated with cell wall-anchoring (Lofling et al., 2011). LPXTG-containing proteins are covalently attached to the cell wall, and at least some of these proteins have been identified as multi-functional proteins (Yamaguchi et al., 2008; Uchiyama et al., 2009; Dalia et al., 2010; Lofling et al., 2011). Our evolutionary analysis indicated that the *nanA* and *bgaA* genes are under considerable negative selection pressure. The *nanA* gene encodes pneumococcal cell surface-localized exo-α-sialidase (NanA) that hydrolyzes α2-3-, α2-6-, and α2-8-linkages of *N*-acetylneuraminic acid residues, and *bgaA* encodes exo-β-galactosidase (BgaA) that hydrolyzes β1-4-linkages of galactose residues, respectively (Hobbs et al., 2018). These glycosidases contribute to biofilm formation in glucose-poor but galactose-rich environments such as the mouse nasopharynx (Blanchette et al., 2016). The glycosidases also disrupt complement deposition and reduce opsonophagocytic killing through catalytic activities (Dalia et al., 2010). In mouse intravenous infection, NanA contributes to pneumococcal invasion into the host central nervous system by aiding penetration through the blood–brain barrier (Uchiyama et al., 2009). However, whether BgaA functions as a virulence factor *in vivo* remains unknown. Thus, we performed a phylogenetic analysis and mouse intravenous infection assay to address this question.

## Materials and Methods

### Phylogenetic and Evolutionary Analyses

The tBLASTn search was used to identify homologs and orthologs of genes that encode cell wall-anchoring proteins (Gertz et al., 2006). Phylogenetic and evolutionary analyses were performed as previously described, with minor modifications (Yamaguchi et al., 2016, 2017, 2019a). Briefly, the sequences were aligned by codon using Phylogear2 (Tanabe, 2008), MAFFT v.7.221 with an L-INS-i strategy (Katoh and Standley, 2013), and Jalview (Waterhouse et al., 2009). Conserved common codons were used for further phylogenetic analysis. The best-fitting codon evolutionary models for MrBayes and RAxML analyses were determined using Kakusan4 (Tanabe, 2011). Bayesian Markov chain Monte Carlo analyses were performed using MrBayes v.3.2.5 or v.3.2.6 (Ronquist et al., 2012), and 2–8 × 10^6^ generations were sampled. To validate phylogenetic inferences, maximum likelihood phylogenetic trees with bootstrap values were generated with RAxML v.8.1.20 (Stamatakis, 2014). Phylogenetic trees were visualized using FigTree v.1.4.4 (Rambaut, 2018). Evolutionary analyses were performed based on aligned orthologous regions of genes that encode cell wall-anchoring proteins and Bayesian phylogenetic trees with a two-rate fixed-effects likelihood function in the HyPhy software package (Pond et al., 2005). For the evolutionary analyses, the level of statistical significance was set at *P* < 0.1 with the HyPhy default setting.

TIGR4 BgaA protein structure was visualized using PyMOL 2.4^1^. The PDB ID is 4CU6 (Singh et al., 2014). The domain structures were identified using MOTIF and Pfam (Kanehisa and Goto, 2000; Finn et al., 2014).

### Bacterial Strains and Construction of Mutant Strains

*S. pneumoniae* strains were cultured at 37°C in Todd-Hewitt broth (BD Biosciences, Franklin Lakes, NJ, United States) supplemented with 0.2% yeast extract (THY; BD Biosciences). Spectinomycin (Wako Pure Chemical Industries, Osaka, Japan) was added to the medium to a concentration of 120 μg/mL for mutant selection and maintenance. The *S. pneumoniae* TIGR4 isogenic *bgaA* (Δ*bgaA*) mutant strain was generated as previously described (Mori et al., 2012; Yamaguchi et al., 2019b). Briefly, the upstream region of *bgaA*, an *aad9* cassette, and the downstream region of *bgaA* were combined by PCR using the primers summarized in Supplementary Table 1. The PCR product was transformed with synthesized CSP2 to construct the mutant strains by double-crossover recombination (Bricker and Camilli, 1999). The mutation was confirmed by site-specific PCR with isolated genomic DNA from the mutant strains. For growth measurement, overnight cultures of each strain were back-diluted 3:100 into fresh THY and grown at 37°C. Growth was monitored by measuring the OD_600_ values every 30 min. The starting point was set at an OD_600_ value of approximately 0.1. The experiment was repeated three times and the data is provided as Supplementary Data 1.

### Mouse Intravenous Infection Assays

All mouse experiments were conducted in accordance with animal protocols approved by the Animal Care and Use Committee of Osaka University Graduate School of Dentistry (28-002-0). The mouse infection assay was performed as previously described (Hirose et al., 2018; Yamaguchi et al., 2019a). Briefly, CD-1 mice (Slc:ICR, 6 weeks, female) were infected by tail vein injection with 1 × 10^6^ CFUs of *S. pneumoniae*. Mouse survival was checked twice daily for 14 days. The experiment was repeated three times and the data is provided as Supplementary Data 2. The pooled data for the three experiments was compared using a log-rank test. Statistical analysis was performed using Prism v.7.0d or v.8.4.2 software (GraphPad, Inc., La Jolla, CA, United States). The level of significance for differences between groups was set at *P* < 0.05.

## Results

### Evolutionary Selective Pressures on Genes Encoding Cell Wall-Anchoring Proteins

**FIGURE 1:**
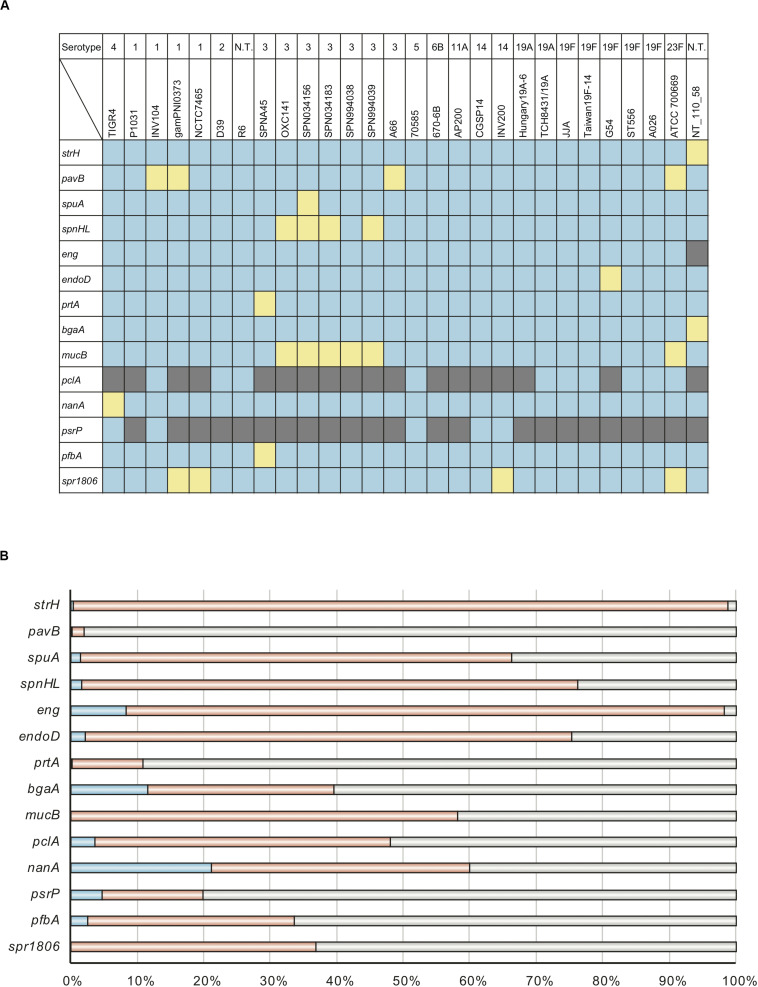
Distribution of genes that encode cell wall-anchoring proteins and percentage of codons that are evolutionarily conserved for these genes. **(A)** Distribution of genes that encode cell wall-anchoring proteins from pneumococcal strains. The gene locus tag numbers are summarized in Supplementary Table 2. Blue, yellow, and gray represent the presence, pseudogenization, and absence of genes, respectively. **(B)** Codons of genes encoding cell wall-anchoring proteins evolving under purifying selection were identified using HyPhy software with phylogenetic trees and aligned sequences. Blue, orange, and gray represent the percentage of codons under purifying selection, comparable common codons, and incomparable codons, respectively. The actual numbers and other parameters are listed in Table 1.

The tBLASTn function was used to search pneumococcal genomes for genes encoding cell wall-anchoring proteins (Figure 1 and Supplementary Table 2). While no genes were found to be conserved as intact open reading frames in all strains, all genes, save for *pclA* and *psrP*, were present in most strains. In particular, although the *nanA* gene in the TIGR4 strain contains a frameshift mutation in C-terminal region, TIGR4 NanA shows sialidase activity, and is assumed to be secreted into milieu instead of being anchored to the cell wall (Gut et al., 2011).

**TABLE 1:** Evolutionary analyses of genes that encode cell wall-anchoring proteins.

**Genes**	**Number of sequences^1^**	**dN/dS**	**Coverage of comparable codons relative to whole protein in TIGR4**	**Codons evolving under positive selection**	**Codons evolving under purifying selection**	**% of codons under purifying selection relative to total codons**
*strH*	23	0.561	98.781%(1297/1313)	0.154%(2/1297)	0.463%(6/1297)	0.457%
*pavB*	25	0.181	1.981%(17/858)	0%(0/17)	11.765%(2/17)	0.233%
*spuA*	22	0.151	66.354%(850/1281)	0%(0/850)	2.353%(20/850)	1.561%
*spnHL*	24	0.343	76.289%(814/1067)	0.369%(3/814)	2.211%(18/814)	1.687%
*Eng*	20	0.145	98.303%(1738/1768)	0.230%(4/1738)	8.458%(147/1738)	8.314%
*endoD*	23	0.243	75.361%(1251/1660)	0.080%(1/1251)	2.878%(36/1251)	2.169%
*prtA*	21	0.013	10.836%(232/2141)	0%(0/232)	3.017%(7/232)	0.327%
*bgaA*	22	0.185	39.526%(883/2234)	0.113%(1/883)	29.332%(259/883)	11.594%
*mucB*	16	1.147	58.128%(118/203)	0%(0/118)	0%(0/118)	0.000%
*pclA*	6	0.328	48.002%(1225/2552)^2^	0.082%(1/1225)	7.592%(93/1225)	3.644%
*nanA*	23	0.170	60.020%(599/998)^2^	0.334%(2/599)	35.392%(212/599)	21.242%
*psrP*	5	0.203	19.845%(948/4777)	0.633%(6/948)	24.367%(231/948)	4.836%
*pfbA*	19	0.423	33.568%(238/709)	0%(0/238)	7.983%(19/238)	2.680%
*spr1806*	19	5.172	36.937%(82/222)	0%(0/82)	0%(0/82)	0.000%

*^1^Sequences with 100% identity were considered to be the same sequence; ^2^compared to D39. Evolutionary analysis was performed by Bayesian inference of aligned sequences from complete genomes of *S. pneumoniae* using the two-rate fixed-effects likelihood function in the HyPhy software package. dN/dS is the ratio of non-synonymous to synonymous changes overall in analyzed genes. Individual codons with a statistically significant signature were also tallied and are expressed as a percentage of the total number of codons included in the analysis.*

To evaluate the degree of evolutionary conservation in cell wall-anchoring proteins, we performed molecular evolutionary calculations based on each phylogenetic relationship and the DNA sequences aligned by codon. The calculated selective pressure for each gene is summarized in Table 1. The percentage of codons that are negatively selected for is visualized in Figure 1B. There was negative selection for over 11% of total codons in *nanA* and *bgaA*. This contrasts with less than 5% of total codons in most other genes, indicating that these two genes play an important role in the success of *S. pneumoniae* species. This same tendency was observed in our previous analysis on pneumococcal CBPs using the same genome sequences. Specifically, in the previous study, more than 13% of codons in the top two genes, *cbpJ* and *lytA*, were negatively selected (Yamaguchi et al., 2019a). Further, we previously reported that the *pfbA* gene showed high specificity to *S. pneumoniae* species and had a low level of sequence diversity (Yamaguchi et al., 2019b). Interestingly, our evolutionary analysis also indicated that the *pfbA* gene is under relaxed selective pressure.

### Phylogenetic Relationships of the *bgaA* Gene

**FIGURE 2:**
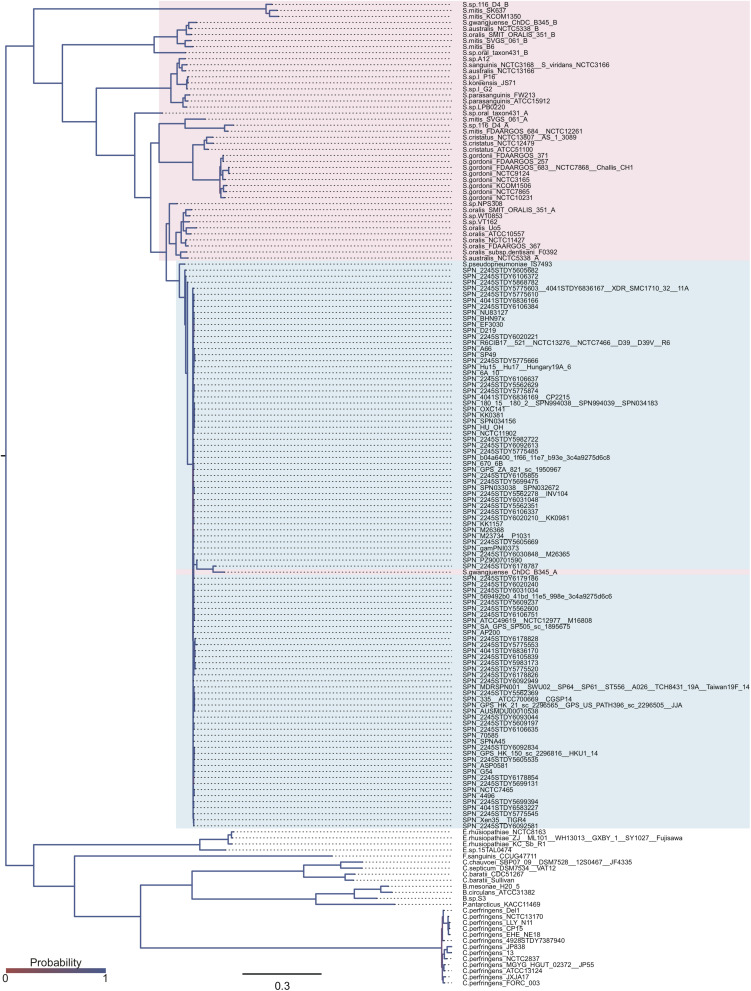
Bayesian phylogenetic analysis of the *bgaA* gene. The codon-based Bayesian phylogenetic relationship was calculated using the MrBayes program. Strains with identical sequences are listed on the same branch. *S. pneumoniae* and *S. pseudopneumoniae bgaA* genes are shaded in cyan. Other streptococcal *bgaA* ortholog genes are shaded in magenta. The color gradation of phylogenetic tree represents posterior probability. The scale bar indicates nucleotide substitutions per site.

Evolutionary analysis indicated that the top two genes, *nanA* and *bgaA*, had high percentage of codons that were under negative selection pressure. We have previously reported that streptococcal *nanA* orthologs diverged into two major groups, with one group consisting of *Streptococcus mitis, Streptococcus intermedius* and *S. pneumoniae*, and the other consisting of *Streptococcus agalactiae* and the *Streptococcus iniae* groups (Yamaguchi et al., 2016). However, the phylogenetic relationships of *bgaA* have not been previously described. We used tBLASTn to search for *bgaA*-homologs, and performed Bayesian and maximum likelihood phylogenetic analyses (Figure 2 and Supplementary Figure 1). The tBLASTn search of the NCBI Nucleotide collection database showed that the *bgaA* gene homologs were identified in various streptococcal species and other Gram-positive bacteria of the phylum Firmicutes, including genus *Clostridium* and *Bacillus.* The β-galactosidase genes of these Gram-positive bacteria were used to root the phylogenetic trees. Bayesian and maximum likelihood phylogenetic analyses produced almost identical trees. The *bgaA* genes in *S. pneumoniae* and *S. pseudopneumoniae* had small genetic distances and formed an independent cluster within a cluster of streptococcal strains. In contrast, orthologous genes in other streptococcal species were genetically diverse. One of the *bgaA* orthologs from the *Streptococcus gwangjuense* strain ChDC B345 belonged to the pneumococcal clusters. As this gene is distinct from other *S. gwangjuense* genes, there is a possibility that it had been obtained from *S. pneumoniae via* horizontal gene transfer.

### Evolutionarily Conserved Catalytic Residues Contribute to the Conformation of BgaA Active Site

**FIGURE 3:**
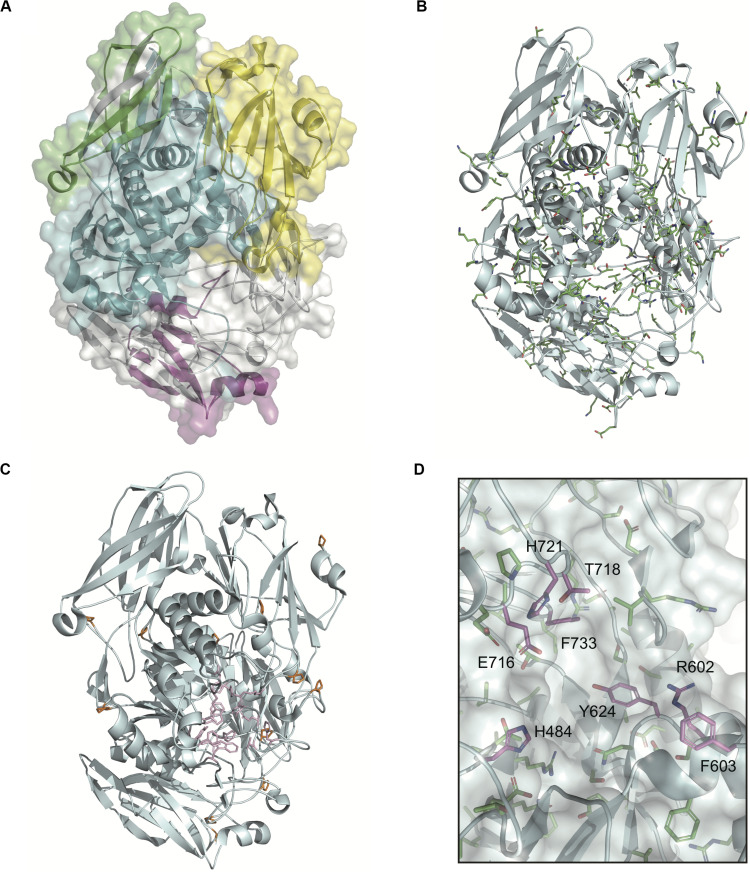
Evolutionarily conserved residues in the BgaA catalytic region. **(A)** Pictorial representation of the structure of TIGR4 BgaA catalytic region (PDB ID: 4CU6). Glycosyl hydrolase family 2 sugar binding domain, glycosyl hydrolase family 2 domain, glycosyl hydrolase family 2 TIM barrel domain, and DUF4982 are colored in yellow, green, cyan, and magenta, respectively. **(B)** The side chains of evolutionarily conserved residues are shown as colored stick models. Carbon, nitrogen, and oxygen are shown as green, blue, and red, respectively. **(C)** BgaA active site is shown as pink stick models, and evolutionary conserved proline residues are in orange. **(D)** The active site of BgaA. The carbon of the evolutionarily conserved catalytic residues is shown in purple (H484, R602, F603, Y624, E716, T718, H721, and F733). The carbon of the evolutionarily conserved non-catalytic residues is shown in green. Nitrogen and oxygen are shown as blue and red, respectively.

The domain structures and amino acid residues of TIGR4 BgaA that are under negative selection are shown in Figures 3A–D and Supplementary Figure 2. BLAST search showed that the TIGR4 BgaA amino acid sequence did not have high similarity with human proteins (Supplementary Table 3). BgaA contains glycosyl hydrolase domains in its N-terminus, and most evolutionarily conserved residues were present, from the glycosyl hydrolase sugar binding domain to the first bacterial Ig-like domain (Figures 3A,B and Supplementary Figure 2). All evolutionarily conserved proline located on loop regions (Figure 3C). Since proline has restricted phi-psi space that arise from the 5-membered ring and stabilize protein structure (Morris et al., 1992), the residues may contribute to the conservation of BgaA catalytic region via stabilization of those loop structures. The active site of BgaA contains 15 catalytic residues, R288, H450, H484, E564, D599, R602, F603, Y624, W685, W708, Y713, E716, T718, H721, and F733 (Singh et al., 2014). All catalytic residues were present in comparable residues encoded by commonly conserved codons in pneumococcal species. Interestingly, eight catalytic residues shown in Figure 3D were evolutionarily conserved ones. The other seven catalytic residues were flanked (R288, H450, W685, and W708) by or located (E564, D599, and Y713) within two residues from evolutionarily conserved residues. These results indicated that evolutionarily conserved residues contribute to the conformation of the BgaA active site.

### BgaA Deficiency Decreases Pneumococcal Pathogenicity in a Mouse Sepsis Model

**FIGURE 4:**
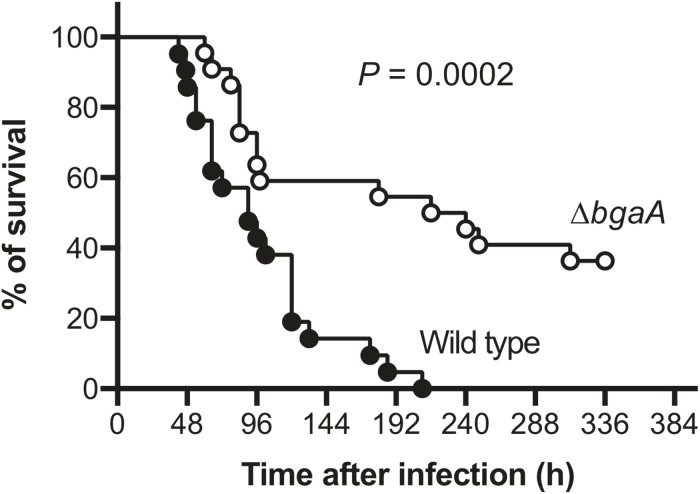
Deficiency of *bgaA* decreases pneumococcal virulence in a mouse model of sepsis. Mice were infected by intravenous injection with 1 × 10^6^ colony forming units (CFU) of *S. pneumoniae* TIGR4 wild type (*n* = 21), or Δ*bgaA* (*n* = 22), and survival was monitored for 14 days. The difference between infected mouse groups was compared using a log-rank test. The data were obtained from three independent experiments.

NanA has been identified as a multi-functional virulence factor. It contributes to pneumococcal biofilm formation, adhesion to and invasion of host epithelial and endothelial cells, inducing excessive host inflammatory responses, and resistance to opsonophagocytosis (King et al., 2006; Uchiyama et al., 2009; Dalia et al., 2010; Chang et al., 2012; Blanchette et al., 2016). BgaA also has been reported to contribute to pneumococcal adherence to host epithelial cells, host immune evasion, and *in vivo* biofilm formation (Dalia et al., 2010; Limoli et al., 2011; Singh et al., 2014; Blanchette et al., 2016). However, several studies reported that deletion of *bgaA* had limited effects on *S. pneumoniae* in mouse colonization models (King et al., 2006; Limoli et al., 2011; Blanchette et al., 2016; Hobbs et al., 2018). It also remains unclear if the deletion of *bgaA* would significantly affect host survival rate in a mouse model of sepsis. Thus, we constructed a TIGR4 *bgaA* mutant (Δ*bgaA*) strain, and performed a mouse intravenous infection assay to compare host survival for TIGR4 wild type and Δ*bgaA* strains. These strains showed similar growth rates in THY medium (Supplementary Figure 3 and Supplementary Data 1). In this infection model, we found that Δ*bgaA*-infected mice had a significantly higher survival rate compared to mice infected with the TIGR4 wild type strain (Figure 4 and Supplementary Data 2). This result indicates that BgaA functions as a virulence factor in a mouse sepsis model.

## Discussion

In this study, we investigated the percentage of codons in genes that encode cell wall-anchoring proteins that are under negative selection in *S. pneumoniae* species. Over 11% of codons in the *nanA* and *bgaA* genes had significant negative selection. As NanA is a well-studied virulence factor in *S. pneumoniae*, we chose to focus on the *bgaA* gene that encodes β-galactosidase. Phylogenetic analysis indicated that the *bgaA* genes in *S. pneumoniae* and *S. pseudopneumoniae* had small genetic distances and formed a distinct cluster within streptococcal *bgaA* orthologs. In addition, eight catalytic residues were evolutionarily conserved, and the other seven catalytic residues were located near evolutionarily conserved ones. We also demonstrated using a mouse intravenous infection model that BgaA contributes to pneumococcal pathogenesis *in vivo*. In combination with our previous work on pneumococcal CBPs, these results suggest that the degree of evolutionary conservation could be an effective parameter for estimating the importance of bacterial cell surface proteins (Yamaguchi et al., 2019a).

While a classical molecular microbiology approach involves analysis of a few representative strains, an evolutionary analysis approach is able to reflect importance in a species based on analysis of a few dozen to 1000s of genome sequences. For genes that encode pneumococcal CBPs, we have recently shown that more than 13% of codons in *cbpJ* and *lytA* genes are negatively selected, and CbpJ acts as a novel virulence factor in pneumococcal pneumonia both *in vitro* and *in vivo* (Yamaguchi et al., 2019a). In this study, we observed negative selective pressure for over 11% of codons in *nanA* and *bgaA*. In contrast, this figure was 3% of codons for *pfbA*. We have previously reported that PfbA is pneumococcal cell surface protein that forms a right-handed parallel β-helix and interacts with human fibronectin, plasmin, plasminogen, albumin, hemoglobin, and fibrinogen (Yamaguchi et al., 2008; Beulin et al., 2014, 2017; Radhakrishnan et al., 2018; Sankar et al., 2020). A BLAST search and phylogenetic analysis showed that *pfbA* is highly conserved in *S. pneumoniae* among mitis group *Streptococcus* (Yamaguchi et al., 2019b). In addition, *in vitro* assays revealed that PfbA functions as an adhesin and invasin for human epithelial cells, is a TLR2 ligand, and an anti-phagocytic factor for human neutrophils (Yamaguchi et al., 2008, 2019b). On the other hand, the deletion of *pfbA* in *S. pneumoniae* had no significant effect on host mortality in a mouse pneumonia model, and enhanced pneumococcal pathogenesis in a mouse model of sepsis (Yamaguchi et al., 2019b). With regards to pneumococcal cell surface proteins, its degree of evolutionary conservation shows good correlation with results in a mouse infection model. Although an evolutionary analysis approach has some limitations, including the fact that it is unable to identify diverse virulence factors within a species (Yamaguchi et al., 2019a), this approach could also be an effective alternative strategy for identification of common virulence factors. Furthermore, in this study, evolutionary analysis with protein structure information open the possibility that the residues under negative selection are important for the protein function and/or structure. Increasing availability and accessibility of bacterial genomic information would allow us to combine evolutionary analysis and laboratory-based approaches to study various bacterial species and proteins.

BgaA is regarded as a multi-functional protein and putative virulence factor; however, BgaA plays a limited or inconsequential role in *in vivo* colonization (King et al., 2006; Burnaugh et al., 2008; Dalia et al., 2010; Limoli et al., 2011; Singh et al., 2014; Blanchette et al., 2016; Hobbs et al., 2018). Here, we revealed that *bgaA* deficiency significantly reduced mortality in a mouse model of blood infection. As the host bloodstream is a glucose-rich environment, the ability to utilize host galactose as an alternative carbon source would not be crucial to pneumococcal survival (Blanchette et al., 2016). BgaA inhibits complement deposition, and consequently opsonophagocytosis, by cleaving *N*-glycans on host glycoproteins that are involved in the complement cascade (Dalia et al., 2010). As such, our observation that the Δ*bgaA* strain reduced host mortality in a mouse blood infection model may be explained by an inability to evade host complement deposition and phagocytosis. Although NanA, BgaA, and StrH have been reported to inhibit C3b deposition to the same extent (Dalia et al., 2010), the evolutionary approach indicated that high rates of *nanA* and *bgaA* codons are under negative selection, while *strH* is not. This discrepancy may arise from specificities of the glycosidases. For example, *N*-linked glycans are cleaved by NanA, BgaA, and StrH sequentially. NanA cleaves terminal sialic acids, after which BgaA cleaves terminal, or NanA-exposed galactose, since sialic acids are commonly linked to the C-3 or C-6 positions of galactose. Subsequently, StrH reportedly cleaves host glycans after NanA and BgaA cleavage (Hobbs et al., 2018). Therefore, one possible hypothesis is that the cleavage order of these glycosidases affects their relative importance. Specifically, StrH may function as a complementary glycosidase since NanA and BgaA may provide sufficient sialic acid, and galactose as alternative carbon sources in glucose-poor environments. Alternatively, the additional roles of NanA and BgaA may simply be more important for pneumococcal survival in the host. However, further studies are required to elucidate the precise role of BgaA in sepsis.

Although, in the current study, we focused on the top two evolutionary conserved genes, the third gene, *eng*, may also serve as an important virulence factor since 8% of *eng* codons are negatively selected. The *eng* gene encodes Eng, endo-α-*N*-acetylgalactosamidase, which specifically cleaves core-1 *O*-linked glycans (Brooks and Savage, 1997; Marion et al., 2009). Although Eng reportedly contributes to the colonization of mouse airway, its specific role in pneumococcal pathogenesis, and whether it affects host mortality, remain unclear. Hence, *eng* is also an attractive target for further investigations.

Emerging antimicrobial resistance of *S. pneumoniae* and serotype replacement after the introduction of pneumococcal vaccines are serious global challenges (O’Neill, 2016; CDC, 2019). A potential solution would involve developing a novel vaccine with a common antigen. *S. pneumoniae* has the ability to import genes from related species and undergo recombination (Kilian et al., 2014; Kilian and Tettelin, 2019). Thus, to minimize the possibility of selective pressure-mediated antigenic variation, a multi-valent vaccine would be superior to a monovalent vaccine. Our evolutionary analysis showed that four pneumococcal cell surface proteins are evolutionarily conserved. A combination of intact or truncated LytA, CbpJ, NanA, and BgaA may be attractive antigen candidates for the development of a universal pneumococcal vaccine. Several groups have already reported that individually, NanA and LytA work as protective antigens in mouse infection models (Berry et al., 1989; Lock et al., 1992; Long et al., 2004; Tong et al., 2005; Yuan et al., 2011; Janapatla et al., 2018). On the other hand, as non-pathogenic native microflora and other *S. mitis* group species also contain some of these genes, it is necessary to examine the possibility that the vaccine may alter the composition of oral and/or lung microbiomes. Evaluation of potential side effects on host microbiome, alongside characterization of the immunogenicity of antigens and efficacy, would help to guide the design of a novel vaccine.

PspA is a promising vaccine candidate for pneumococcal infection, as this vaccine is generally assumed to be multi-valent since PspA is a highly variable protein under positive selection (Piao et al., 2014; Chen et al., 2015; Cornick et al., 2017; Yamaguchi et al., 2019a). Hence, host immune systems, including the humoral immune response, would select for pneumococcal PspA. This may prove advantageous for a vaccine candidate since the presence of positive selection indicates that the protein is capable of inducing host acquired immunity. At the same time, the variety of PspA reflects that *S. pneumoniae* evolves to evade host acquired immunity through obtaining PspA mutations. Thus, the introduction of PspA vaccine would induce novel selective pressure causing “PspA replacement,” referring to an increase in vaccine-uncovered type PspA, as well as serotype replacement by pneumococcal polysaccharide capsule vaccines. It would, therefore, be of importance to determine whether the multi-valent PspA vaccine effectively covers the selective pressure-extended PspA variety. Indeed, two different selective pressures for polysaccharide and PspA vaccines may overcome the replacement issue. Hence, during pandemics of emerging infectious diseases, such as COVID-19, an effective strategy may be to develop novel vaccines that simultaneously target conserved proteins under positive and negative selections. However, proteins under positive selection are suitable as initial targets for rapid vaccine development as they have a strong probability to elicit antibodies in humans. Meanwhile, to address the evolution of pathogens, evolutionarily conserved proteins would serve as effective targets in the development of later vaccine candidates.

## Data Availability Statement

The raw data supporting the conclusions of this article will be made available by the authors, without undue reservation.

## Ethics Statement

The animal study was reviewed and approved by the Animal Care and Use Committee of Osaka University Graduate School of Dentistry (28-002-0).

## Author Contributions

MY and SK designed the study. MY and KH performed bioinformatics analyses. MT performed the animal experiments. MY, KG, YH, TS, MN, and SK contributed to the experimental setup. MY wrote the manuscript. MT, KH, KG, YH, TS, MN, NU, and SK contributed to the writing of the manuscript. All authors contributed to the article and approved the submitted version.

## Conflict of Interest

The authors declare that the research was conducted in the absence of any commercial or financial relationships that could be construed as a potential conflict of interest.

## Footnotes

1 https://pymol.org/2/
