# Supplementary material for: Role of BgaA as a Pneumococcal Virulence Factor Elucidated by Molecular Evolutionary Analysis
Source: Front Microbiol. 2020 Sep 24;11:582437. doi: 10.3389/fmicb.2020.582437 (PMC7541833; doi:10.3389/fmicb.2020.582437)
Supplement: Supplementary file 1 [file Data_Sheet_1.PDF]

Supplementary Table 1. Primers used in this study

Supplementary Table 2. Locus tag number of genes encoding cell wall-anchoring proteins in each pneumococcal strain.

Supplementary Table 3. BLASTP results: Sequences of *Homo sapiens* producing significant alignments with TIGR4 BgaA

Supplementary Figure 1. Maximum likelihood phylogenetic analysis of the *bgaA* gene.

The codon-based maximum likelihood phylogenetic relationship was calculated using the RAxML program. Strains with identical sequences are listed on the same branch. *S. pneumoniae* and *S. pseudopneumoniae bgaA* genes are shaded in cyan. Other streptococcal *bgaA* orthologue genes are shaded in magenta. The color gradation represents bootstrap value. The scale bar indicates nucleotide substitutions per site.

Supplementary Figure 2. Amino acid sequences and domain structures of BgaA in strain TIGR4.

Bold, black underlined, and magenta underlined characters represent comparable codons and the codons under purifying or positive selection, respectively. The motifs and domains are colored as indicated.

Supplementary Figure 3. Growth of *S. pneumoniae* TIGR4 WT and Δ*bgaA* strains. *S. pneumoniae* strains were grown in THY broth at 37°C. Data are presented as the mean of six samples from a representative experiment. S.D. values are represented by vertical lines.

Supplementary Table 1. Primers used in this study

Primers	Sequence (5' to 3')
T4bgaAKOuF	tatgactggagtatcggatatatttgaatacactacgaaca
T4bgaAKOuR	tacttgattgttttctcaatttttttataattttttttaat
T4bgaAKOaF	gtaagtttgctgtgggagcttgct
T4bgaAKOaR	tattcaaatatatccgatactccagtcatacggt
T4bgaAKOdF	ttataaaaaaattgagaaaacaatcaagtagcttcaac
T4bgaAKOdR	ggcttgccgcactacctgtacttgg

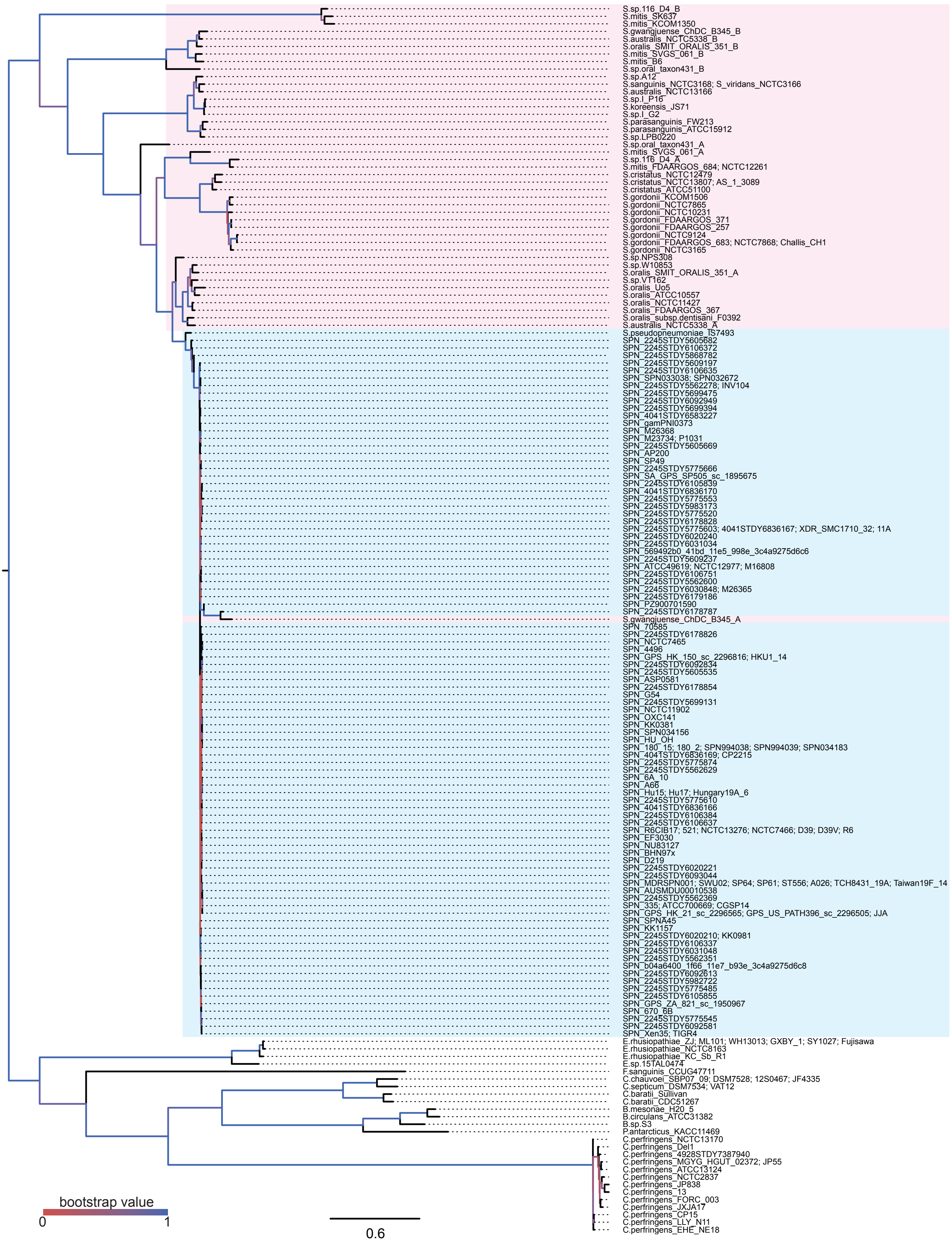

Supplementary Figure 1. Yamaguchi *et al.*

MGKGHWNRKRVYSIRKFAVGACSVMIGTCAVLLGGNIAGESVVYADETLITHTAEKPKEEKMIVEEKADKALETK
NIVERTEQSEPSSTEAIASEKKEDEAVTPKEEKVSAKPEEKAPRIESQASNQEKPLKEDAKAVTNEEVNQMIEDR 150
KVDFNQNWYFKLNANSKEAIKPDADVSTWKKLDLPYDWSIFNDFDHESPAQNEGGQLNGGEAWYRKTFKLDEKDL
KKNVRLTFDGVYMDSQVYVNGQLVGHYPNGYNQFSYDITKYLQKDGRENVIAVHAVNKQPSSRWYSGSGIYRDVT 300
LQVTDKVHVEKNGTTILTPKLEEQQHGKVETHVTSKIVNTDDKDHELVAEYQIVERGGHAVTGLVRTASRTLKAH
ESTSLDAILEVERPKLWTVLNDKPALYELITRVYRDGQLVDAKKDLFGYRYYHWTPNEGFSLNGERIKFHGVSLH 450
HDHGALGAEENYKAEYRRLKQMKEMGVNSIRTTHNPASEQTLQIAAELGLLVQEEAFDTWYGGKKPYDYGRFFEK
DATHPEARKGEKWSDFDLRTMVERGKNNPAIFMWSIGNEIGEANGDAHSLATVKRLVKVIKDVDKTRYVTMGADK 600
FRFGNGSGGHEKIADELDAVGFNYSEDNYKALRAKHPKWLIYGSETSSATRTRGSYYRPERELKHSNGPERNYEQ
SDYGNDRVGWGKTATASWTFDRDNAGYAGQFIWTGTDYIGEPTPWHNQNQTPVKSSYFGIVDTAGIPKHDFYLYQ 750
SQWVSVKKKPMVHLLPHWNWENKELASKVADSEGKIPVRAYSNASSVELFLNGKSLGLKTFNKKQTSDGRTYQEG
ANANELYLEWKVAYQPGTLEAIARDESGKEIARDKITTAGKPAAVRLIKEDHAIAADGKDLTYIYYEIVDSQGNV 900
VPTANNLVRFQLHGQGQLVGVDNGEQASRERYKAQADGSWIRKAFNGKGVAIVKSTEQAGKFTLTAHSDLLKSNQ
VTVFTGKKEGQEKTVLGTEVPKVQTIIGEAPEMPTTVPFVYSDGSRAERPVTWSSVDVSKPGIVTVKGMADGREV 1050
EARVEVIALKSELPVVKRIAPNTDLNSVDKSVSYVLIDGSVEEYEVDKWEIAEEDKAKLAIPGSRIQATGYLEGQ
PIHATLVVEEGNPAAPAVPTVTVGGEAVTGLTSQKPMQYRTLAYGAKLPEVTASAKNAAVTVLQASAANGMRASI 1200
FIQPKDGGPLQTYAIQFLEEAPKIAHLSLQVEKADSLKEDQTVKLSVRAHYQDGTQAVLPADKVTFSTSGEGEVA
IRKGMLELHKPGAVTLNAEYEGAKDQVELTIQANTEKKIAQSIRPVNVVTDLHQEPSLPATVTVEYDKGFPKTHK 1350
VTWQAIPKEKLDSYQTFEVLGKVEGIDLEARAKVSVEGIVSVEEVSVTTPIAEAPQLPESVRTYDSNGHVSSAKV
AWDAIRPEQYAKEGVFTVNGRLEGTQLTTKLHVRVSAQTEQGANISDQWTGSELPLAFASDSNPSDPVSNVNDKL 1500
ISYNNQPANRWTNWNRTNPEASVGVLFGDSGILSKRSVDNLSVGFHEDHGVGVPKSYVIEYYVGKTVPTAPKNPS
FVGNEDHVFNDSANWKPVTNLKAPAQLKAGEMNHFSFDKVETYAVRIRMVKADNKRGTSITEVQIFAKQVAAAKQ 1650
GQTRIQVDGKDLANFNPDLTDYYLESVDGKVPAVTASVSNNGLATVVPSVREGEPVRVIAKAENGDILGEYRLHF
TKDKSLLSHKPVAAVKQARLLQVGQALELPTKVPVYFTGKDGYETKDLTVEWEEVPAENLTKAGQFTVRGRVLGS 1800
NLVAEITVRVTDKLGETLSDNPNYDENSNQAFASATNDIDKNSHDRVDYLNDGDHSENRRWTNWSPTPSSNPEVS
AGVIFRENGKIVERTVTQGKVQFFADSGTDAPSKLVLERYVGPEFEVPTYYSNYQAYDADHPFNNPENWEAVPYR 1950
ADKDIAAGDEINVTFKAIKAKAMRWRMERKADKSGVAMIEMTFLAPSELPQESTQSKILVDGKELADFAENRQDY
QITYKGQRPKVSVEENNQVASTVVDSGEDSFPVLVRLVSESGKQVKEYRIHLTKEKPVSEKTVAAVQEDLPKIEF 2100
VEKDLAYKTVEKKDSTLYLGETRVEQEGKVGKERIFTAINPDGSKEEKLREVVEVPTDRIVLVGTKPVAQEAKKP
QVSEKADTKPIDSSEASQTNKAQLPSTGSAASQAAVAAGLTLLGLSAGLVVTKGKKED*

- YSIRK type signal peptide
- Glycosyl hydrolases family 2, sugar binding domain
- Glycosyl hydrolases family 2
- Glycosyl hydrolases family 2, TIM barrel domain
- Domain of unknown function (DUF4982)
- Bacterial Ig-like domain (group 4)
- LPXTG motif

Supplementary Figure 2. Yamaguchi *et al.*

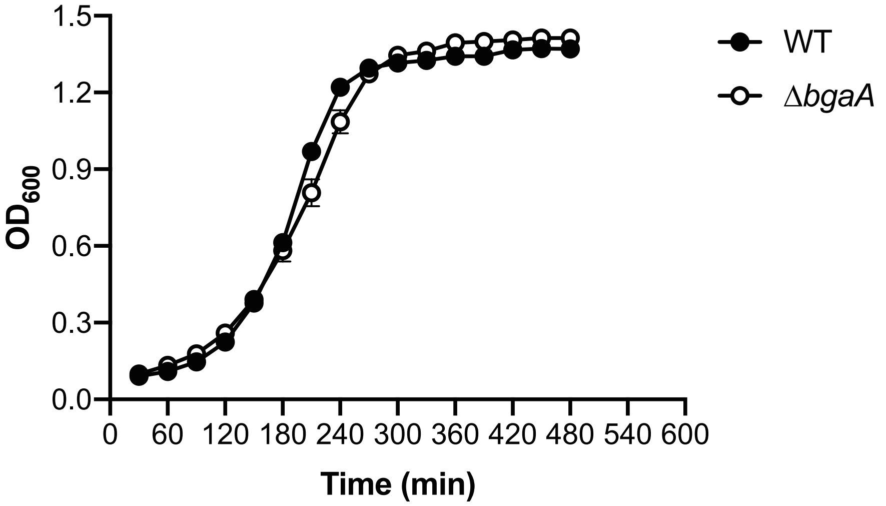

Supplementary Figure 3. Yamaguchi *et al.*
